# Multiple ATR-Chk1 Pathway Proteins Preferentially Associate with Checkpoint-Inducing DNA Substrates

**DOI:** 10.1371/journal.pone.0022986

**Published:** 2011-07-29

**Authors:** Seçil Yilmaz, Aziz Sancar, Michael G. Kemp

**Affiliations:** Department of Biochemistry and Biophysics, University of North Carolina School of Medicine, Chapel Hill, North Carolina, United States of America; University of Minnesota, United States of America

## Abstract

The ATR-Chk1 DNA damage checkpoint pathway is a critical regulator of the cellular response to DNA damage and replication stress in human cells. The variety of environmental, chemotherapeutic, and carcinogenic agents that activate this signal transduction pathway do so primarily through the formation of bulky adducts in DNA and subsequent effects on DNA replication fork progression. Because there are many protein-protein and protein-DNA interactions proposed to be involved in activation and/or maintenance of ATR-Chk1 signaling *in vivo*, we systematically analyzed the association of a number of ATR-Chk1 pathway proteins with relevant checkpoint-inducing DNA structures *in vitro*. These DNA substrates included single-stranded DNA, branched DNA, and bulky adduct-containing DNA. We found that many checkpoint proteins show a preference for single-stranded, branched, and bulky adduct-containing DNA in comparison to undamaged, double-stranded DNA. We additionally found that the association of checkpoint proteins with bulky DNA damage relative to undamaged DNA was strongly influenced by the ionic strength of the binding reaction. Interestingly, among the checkpoint proteins analyzed the checkpoint mediator proteins Tipin and Claspin showed the greatest differential affinity for checkpoint-inducing DNA structures. We conclude that the association and accumulation of multiple checkpoint proteins with DNA structures indicative of DNA damage and replication stress likely contribute to optimal ATR-Chk1 DNA damage checkpoint responses.

## Introduction

Cells are constantly exposed to a variety of endogenous and exogenous agents that form bulky adducts on DNA, including by the environmental carcinogens ultraviolet (UV) light and benzo[a]pyrene. These lesions are problematic because they interfere with many DNA metabolic processes, including transcription and DNA replication. Though these lesions can be removed from the genome through the process of nucleotide excision repair [1], the lack or inefficiency of this repair process may lead to cell death, mutagenesis, or to abnormal cell proliferation.

To combat DNA damage, eukaryotic cells have evolved DNA damage checkpoint responses, which are signal transduction pathways that respond to DNA damage by delaying cell cycle progression to allow time for DNA repair [2], [3]. In organisms ranging from yeast to man, the phosphoinositide-3-kinase-related protein kinase (PIKK) ATR plays a primary role in the initial response to bulky DNA adducts and to problems that arise during replication of adducted bases [4]. A critical substrate of ATR is the signal transducing kinase Checkpoint Kinase 1 (Chk1). Upon phosphorylation and activation by ATR, Chk1 phosphorylates additional protein factors that impact DNA repair and cell cycle progression, such as the protein phosphatase Cdc25A. Phosphorylation of Cdc25A by Chk1 triggers its ubiquitination and degradation by the proteasome, therefore preventing Cdc25A from dephosphorylating and activating the cyclin-dependent kinases (CDKs) that drive cell cycle progression [2]. An additional target of the DNA damage checkpoint in S phase is the replication initiation factor Cdc45, which upon DNA damage is prevented from being loaded at DNA replication origins in an ATR- and Chk1-dependent manner [5].

An essential protein for ATR kinase activation is the ATR-interacting protein ATRIP, which is constitutively bound to ATR and facilitates the recruitment of ATR to DNA [6], [7]. However, there are several proposed mechanisms by which the ATR/ATRIP complex and the ATR-Chk1 pathway may become activated by genotoxic stress. Though the ATR/ATRIP complex may directly sense DNA damage itself [8] or through association with its activator protein TopBP1 [9], [10], a variety of other protein factors are also implicated in the direct recognition of DNA damage and replication stress. These DNA damage “sensor” proteins include a variety of DNA repair factors that directly associate with specific forms of DNA damage, such as bulky DNA adducts, DNA mismatches, interstrand crosslinks, single-stranded DNA (ssDNA), and primer-template junctions. Through additional protein-protein interactions, these repair factors may directly and stably recruit the ATR kinase to the DNA damage site to initiate signaling responses.

Two of the most prominent ATR-mediated DNA damage checkpoint “sensor” proteins include Replication Protein A (RPA) [11], [12], a ssDNA-binding protein that binds the ATR-interacting protein ATRIP to recruit the ATR kinase to sites of DNA damage [7], and the primer-template junction clamp complex Rad9-Hus1-Rad1 (9-1-1), which through a direct protein-protein interaction brings TopBP1 into proximity of ATR to enable full activation of ATR kinase activity [13]. There are also additional factors that may aid the recruitment or activation of ATR at specific forms of DNA base damage, such as the nucleotide excision repair factor XPA [1], the Fanconi Anemia-associated factor FAAP24 [14], [15], and the mismatch repair protein MSH2 [16]–[18].

An additional class of protein factors has been suggested to facilitate the specific phosphorylation of Chk1 or other substrates by ATR in order to amplify or maintain checkpoint signaling responses. These checkpoint “mediator” proteins include the direct ATR kinase-activating protein TopBP1 [19] and the Chk1-interacting factor Claspin [20], [21]. Similarly, based on the ability of the Tipin subunit to directly bind both RPA and Claspin, the Timeless-Tipin complex may mediate Chk1 phosphorylation by ATR at sites of DNA damage and replication stress bound by RPA [22].

Though a great deal of progress has been made in identifying proteins and protein-protein interactions that regulate ATR-Chk1 checkpoint signaling responses, significant questions remain regarding the DNA substrates and protein-DNA interactions that trigger utilization of this signaling pathway. Several recent reports have demonstrated that the artificial tethering of DNA damage checkpoint proteins to DNA is sufficient to induce cell cycle checkpoint responses in the absence of overt DNA damage or replication stress [23]–[25]. Thus, the accumulation or concentration of checkpoint signaling proteins on DNA may be a key component in the activation of DNA damage checkpoint responses. In this report, we therefore systematically characterized the association of a panel of ATR-Chk1 pathway proteins with several of the putative signals of DNA damage and replication stress—single-stranded DNA (ssDNA), branched DNA, and bulky adduct-containing DNA. To varying degrees, we find that many of these pathway proteins show preferential association with these checkpoint-inducing DNA structures. We also find that the ionic strength of the binding reaction can significantly impact checkpoint protein-DNA interactions. Furthermore, we find that under mildly stringent binding conditions, the checkpoint mediator proteins Claspin and Tipin show the greatest ability to discriminate checkpoint-inducing DNA structures from undamaged, double-stranded DNA (dsDNA). We therefore conclude that robust activation of DNA damage checkpoint responses by branched DNA structures and bulky DNA adducts may depend, in part, on the formation of multiple, cooperative checkpoint protein-DNA interactions.

## Methods

### Protein Purification

Each of the following proteins was purified as previous described from either *E. coli* or baculovirus-infected insect cells: RPA [26], FLAG-Rad9-Hus1-Rad1 [27], FLAG-ATRIP [28], GST-TopBP1 [9], [10], [29], FLAG-Claspin [30], Tipin-His [22], FLAG-Timeless [31], FLAG-Timeless/His-Tipin complex [31], His-Chk1 [9], [10], [29], GST-Cdc45 [32], and MBP-XPA [33]. FAAP24-His was cloned from Open Biosystems clone MHS1011-9199435 into pET21b, expressed in BL21 (DE3) cells, and purified with Ni-NTA agarose (Qiagen). Purified proteins were analyzed by SDS-PAGE and coomassie blue staining.

### DNA substrates

The following 50-mer oligonucleotides were used to prepare ssDNA, dsDNA, and branched, fork-like DNA for *in vitro* pull-down assays: Oligo1, 5′-Biotin-GACGCTGCCGAATTCTGGCTTGCTAGGACATCTTTGCCCACGTTGACCCG-3′; Oligo2, 5′-GCGATAGTCTCTAGACAGCATGTCCTAGCAAGCCAGAATTCGGCAGCGTC-3′; Oligo3, 5′-CGGGTCAACGTGGGCAAAGATGTCCTAGCAAGCCAGAATTCGGCAGCGTC. The ssDNA pull-down assays utilized Oligo1. The dsDNA and fork DNA substrates were prepared by annealing Oligo1 with Oligo3 or Oligo2, respectively. The oligonucleotides were prepared and combined in annealing buffer (50 mM Tris pH 7.4, 50 mM NaCl) and heated at 95°C for 5 min in a heat block before slow cooling to room temperature. Annealed oligonucleotides were stored at −20°C until use. DNA substrates were coupled to Dynabeads M-280 Streptavidin (Invitrogen) at a concentration of 0.25 pmol of DNA per µl of magnetic beads according to the manufacturer's instructions. Undamaged and *N*-acetyoxy-2-acetylaminofluorene (AAF)-damaged pUC19 plasmid DNA were prepared and coupled to Dynabeads M-280 Streptavidin at a concentration of 50 ng of DNA per µl of beads as previously described [34].

### Pull-down assays with immobilized DNA

Pull-down assays with immobilized DNAs were performed essentially as previously described [22], [34]. For pull-down assays with ssDNA, dsDNA, and fork DNA, the appropriate DNA-bead complexes (0.5 pmol) was mixed with the indicated amount of protein in 50 µl of binding buffer (10 mM Tris pH 7.4, 50 mM NaCl, 10 µg/ml BSA, 10% glycerol, 0.01% NP-40). Experiments with undamaged and AAF-damaged DNA were performed similarly, except with plasmid DNA-bound beads (50 ng) and binding buffer containing 100 mM NaCl. After incubation for 30 min at room-temperature, the DNA-beads were retrieved with a magnet and washed three times with binding buffer (200 µl). DNA-beads and associated proteins were then boiled in 1× SDS-PAGE sample buffer, separated by SDS-PAGE, and transferred to nitrocellulose. Proteins were detected by immunoblotting using antibodies against either the affinity tag or the native protein, according to standard procedures. Blots were scanned and quantified using ImageQuant v5.0 software. The highest signal on each blot was set to an arbitrary value of 1, and then every other signal was set relative to this value. Results were graphed to show the average and standard deviation from two to four independent experiments.

### Antibodies

Antibodies against RPA70 (sc-28304), MBP (sc-809), and GST (sc-138) were purchased from Santa Cruz Biotechnology. Anti-FLAG antibody (F3165) was from Sigma. Anti-His antibody (AM1010a) was purchased from Abgent.

## Results

### Purification of ATR-Chk1 checkpoint pathway proteins

**Figure 1:**
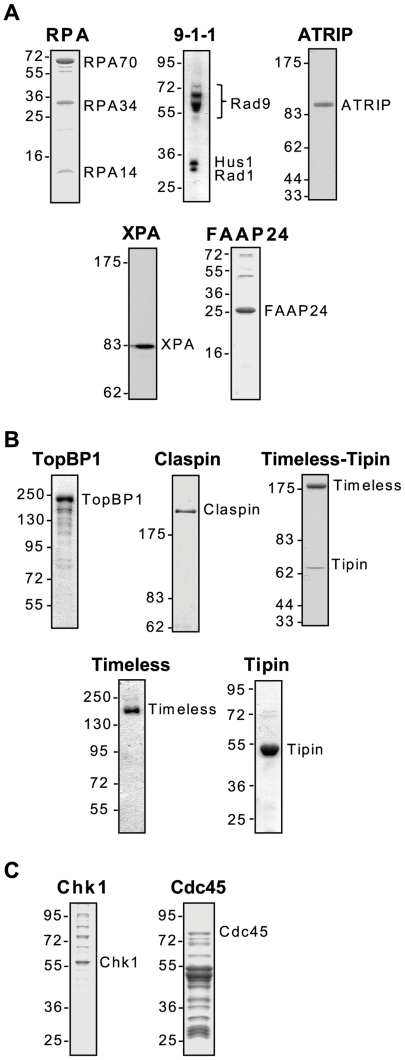
Purification of ATR-Chk1 pathway checkpoint proteins. Recombinant forms of the indicated checkpoint proteins were separated by SDS-PAGE and visualized by coomassie blue staining. Proteins are categorized into (A) DNA damage sensors, (B) mediators, and (C) transducers/effectors.

To study the association of ATR-Chk1 pathway proteins with DNA damage checkpoint-inducing DNA substrates, we first purified several different classes of DNA damage checkpoint proteins (**Figure 1**). This set of proteins includes those factors traditionally referred to as DNA damage “sensors”, based on various biochemical and genetic evidence suggesting that these proteins directly detect specific forms of DNA damage and replication stress in the cell. The DNA damage sensors we purified included the ssDNA-binding protein RPA, the primer-template junction clamp 9-1-1, and the ATR-interacting protein ATRIP, which binds to both RPA and the ATR kinase [7], [35] (Figure 1A). We also purified XPA, an essential component of the nucleotide excision repair machinery that removes bulky DNA adducts from the genome [1], [33]. XPA has been shown to directly interact with ATR [36] and to be required for optimal phosphorylation of Chk1 by ATR after UV irradiation [37]. Similarly, the Fanconi Anemia-associated protein FAAP24 has been suggested to directly detect interstrand crosslinks and to recruit RPA and ATR-ATRIP to induce DNA damage checkpoint responses to interstrand crosslinks [14], [15]. We therefore purified this protein as well to test its association with checkpoint-inducing forms of DNA.

Another class of checkpoint proteins we purified is a group termed “mediators”, due their specific role in facilitating the phosphorylation of Chk1 by ATR. As shown in Figure 1B, these proteins included the following: TopBP1, a direct stimulator of ATR kinase activity [19]; Claspin, which was initially identified as a Chk1-interacting protein [20], [21], [38]; and the Timeless-Tipin complex [31], [39], which through the Tipin subunit directly binds to both RPA and Claspin [22]. Though Timeless-Tipin is a heterodimeric complex *in vivo*, we also purified its individual subunits to investigate whether or not each subunit associates with DNA.

The last set of proteins included factors termed “transducers” and “effectors”. Chk1 is a primary member of this class because, through phosphorylation and activation by ATR, it transduces DNA damage signaling from ATR to target molecules such as Cdc25A [2]. During the intra-S phase checkpoint response, the ATR-Chk1 pathway also targets the replication initiation factor Cdc45 [5]. We therefore also purified this protein to study its DNA binding properties.

Together, this comprehensive set of purified proteins represents factors involved in each step of the ATR-Chk1 signal transduction pathway that responds to bulky DNA damage and replication stress in human cells.

### Association of ATR-Chk1 pathway proteins with ssDNA and fork-like DNA

A primary trigger for ATR activation in response to UV and similar agents that induce formation of bulky adducts in DNA is believed to be ssDNA that is generated after DNA polymerases stall at adducted bases and become uncoupled from DNA helicase activity at the replication fork [4], [40]. We therefore examined the association of each of the proteins shown in Figure 1 with ssDNA, dsDNA, and branched, replication fork-like DNA. The ssDNA was a 50-mer ssDNA and the dsDNA substrate included its complementary DNA sequence. The branched DNA structure contained 31 base pairs of complementary sequence and 19 nt of ssDNA lacking complementarity. Each of these DNA structures included a 5′-biotin on one strand in order to immobilize the DNAs on streptavidin-coated magnetic beads. After titrating increasing amounts of the appropriate test protein into binding reactions with each DNA structure, the DNA-beads were briefly washed and the associated proteins analyzed by SDS-PAGE and immunoblotting.

**Figure 2:**
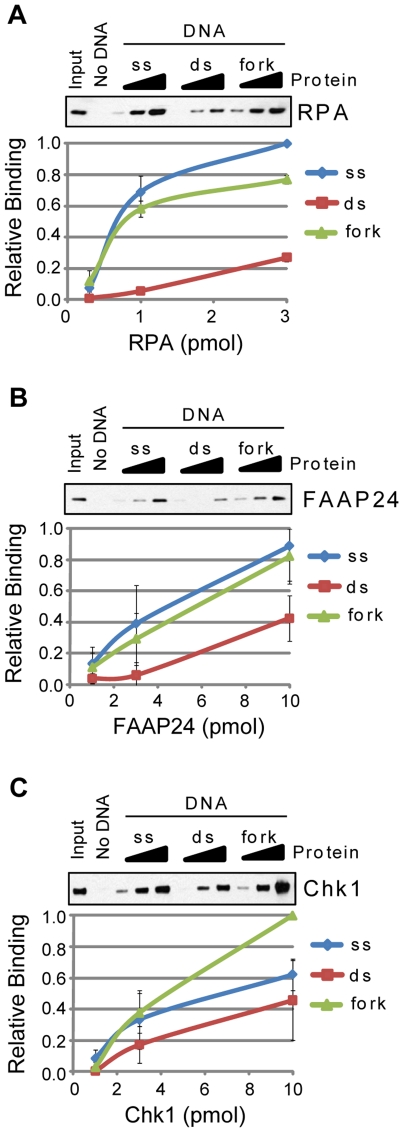
RPA, FAAP24, and Chk1 preferentially bind ssDNA and branched DNA. The association of each of the indicated purified proteins with beads alone (No DNA) or to single-stranded (ss), double-stranded (ds), and branched, replication fork-like (fork) DNA bound to magnetic beads was analyzed by SDS-PAGE and immunoblotting. Each of the following proteins was analyzed for binding to each DNA structure: (A) RPA, (B) FAAP24, and (C) Chk1. Reactions were carried out as described in the Methods section. Experiments were repeated two to four times and the average binding values and standard deviations graphed. Input lane contains the lowest amount of protein used in each binding assay.

As shown in **Figure 2**, several proteins showed greater binding to both ssDNA and the branched, fork-like DNA structure than to double-stranded DNA. Consistent with its role as a ssDNA-binding protein, for example, up to 10-fold more RPA was retained on the immobilized ssDNA and fork-like DNA than on the double-stranded DNA (Figure 2A). The preferential binding of RPA to the fork-like structure was likely due to the affinity of RPA for the ssDNA present in the branched DNA and not due to a specific association with the branched structure itself. Several other factors showed similar binding characteristics, including FAAP24 (Figure 2B) and Chk1 (Figure 2C). These findings indicate that these factors bind ssDNA and likely do not directly “sense” or detect the transition from complementarity to non-complementarity within the branched DNA substrate.

**Figure 3:**
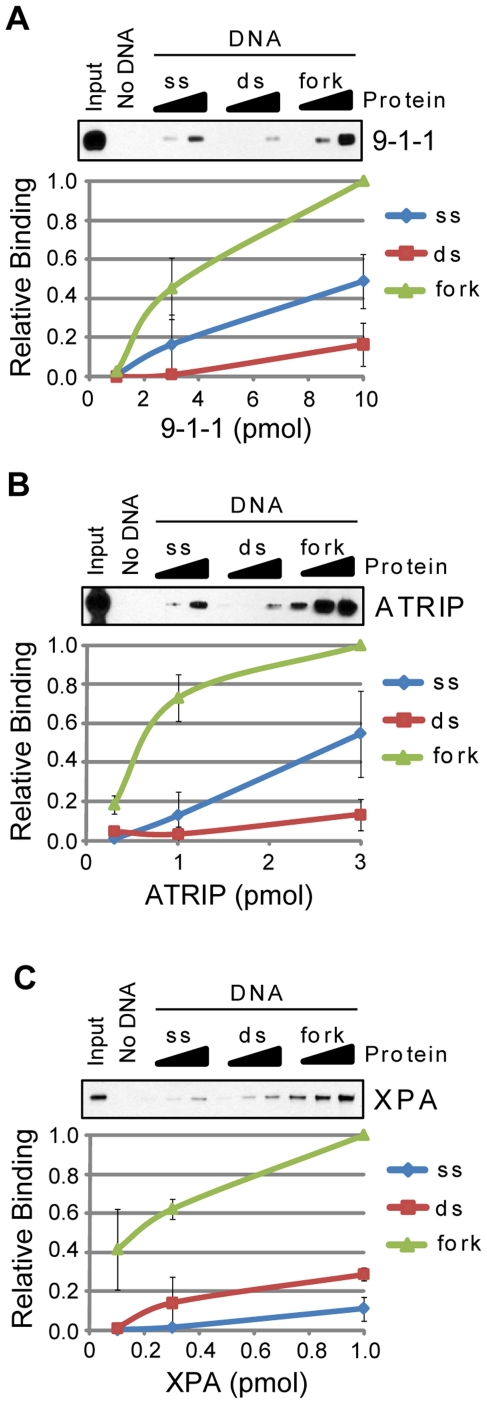
The DNA damage checkpoint sensors 9-1-1, ATRIP, and XPA preferentially bind branched DNA. The association of each of the indicated purified proteins with beads alone (No DNA) or to single-stranded (ss), double-stranded (ds), and branched, replication fork-like (fork) DNA bound to magnetic beads was analyzed by SDS-PAGE and immunoblotting. Each of the following proteins were analyzed for binding to each DNA structure: (A) 9-1-1, (B) ATRIP, and (C) XPA. Reactions were carried out as described in the Methods section.

However, several of the DNA damage “sensor” proteins did show preferential association with the branched DNA in comparison to either ssDNA or dsDNA. This set of proteins is shown in **Figure 3** and includes the 9-1-1 complex (Figure 3A), ATRIP (Figure 3B), and XPA (Figure 3C). Structural and functional data indicate that the 9-1-1 complex associates with primer-template junctions at sites of DNA damage and replication stress [2], [41]. Thus the apparent elevated affinity of 9-1-1 for the fork-like structure may indicate that, within the context of this experiment, 9-1-1 may bind to one of the free ssDNA ends that are present on the branched DNA structure, migrate or slide down the DNA, and then become “trapped” on the DNA at the ssDNA/dsDNA fork junction.

Though the direct contact of ATRIP with RPA stabilizes ATRIP on RPA-coated ssDNA *in vitro* and aids its recruitment to immunofluorescently-defined foci *in vivo* [7], [35], ATRIP can also directly bind to DNA in the absence of RPA [28], [42]. Our finding that ATRIP showed an increased affinity for the branched DNA structure in comparison to either ssDNA or dsDNA (Figure 3B) indicates that ATRIP has the potential to directly sense stalled replication forks *in vivo* in the absence of other factors, including RPA.

XPA has previously been shown to bind branched DNA structures [43], which may be relevant to its coordination of the incision events during nucleotide excision repair [1]. Though XPA directly binds ATR [36] and is required for optimal UV-induced phosphorylation of Chk1 [37], there is currently no evidence that the association of XPA with branched DNA structures is relevant to ATR-Chk1 signaling. These results highlight the fact that only limited conclusions can be drawn from protein-DNA interaction data in the absence of other relevant, biological evidence.

**Figure 4:**
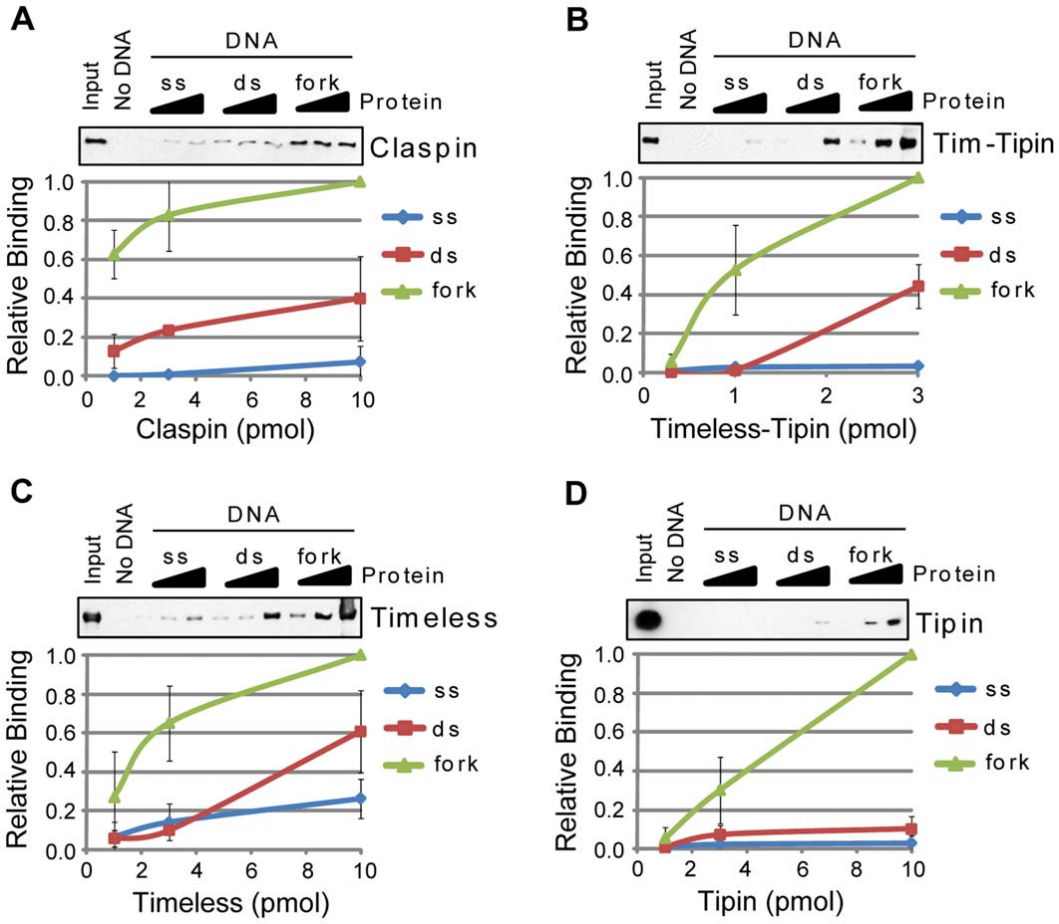
The DNA damage checkpoint mediators Claspin, Timeless, and Tipin preferentially bind branched DNA. The association of each of the indicated purified proteins with no DNA (beads alone), single-stranded (ss), double-stranded (ds), and branched, replication fork-like (fork) DNA was analyzed by SDS-PAGE and immunoblotting. Each of the following proteins was analyzed for binding to each DNA structure: (A) Claspin, (B) Timeless-Tipin (Tim-Tipin) complex, (C) Timeless, and (D) Tipin. Reactions were carried out as described in the Methods section.

Interestingly, Claspin, the Timeless-Tipin complex, and Timeless and Tipin individually all showed preferential association with the branched DNA (**Figure 4**). Analysis of fragments of each of these proteins showed that each protein contains at least one domain that contributes to the overall DNA binding properties of the holoenzyme (**Figure S1**).These results are consistent with the purported role for these proteins as a “replication fork-protection complex” that stabilizes replication forks that stall due to DNA damage or abnormal DNA structures [44]–[46]. Similarly, Claspin and its yeast homolog Mrc1 were previously shown to preferentially associate with branched DNA structures by electrophoretic mobility shift assay and electron microscopy [30], [47], [48]. The preferential association of both the Timeless-Tipin complex and its individual subunits with the branched DNA (Figure 4 B–C) indicates that several protein-DNA interactions may be involved in the binding of the complex to DNA. Though the relative amount of Tipin that associated with the DNA was less than that for Claspin or Timeless, at its highest concentration in the binding reaction, it showed a much greater (>10-fold) preference for the branched DNA in comparison to either the ssDNA or dsDNA (Figure 4D). These results suggest that Tipin may play an important role in detecting branched DNA structures.

**Figure 5:**
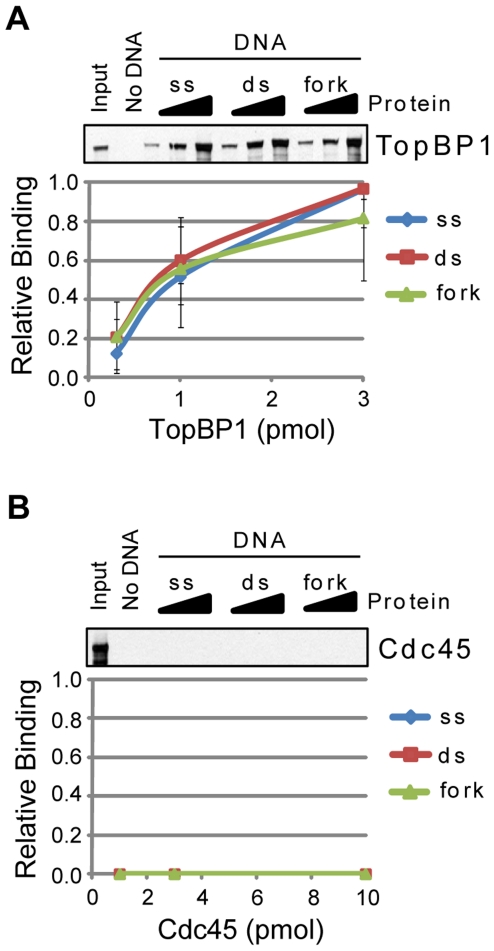
Association of TopBP1 and Cdc45 with DNA. The association of each of the indicated purified proteins with beads alone (No DNA) or to single-stranded (ss), double-stranded (ds), and branched, replication fork-like (fork) DNA bound to magnetic beads was analyzed by SDS-PAGE and immunoblotting. Each of the following proteins was analyzed for binding to each DNA structure: (A) TopBP1 and (B) Cdc45. Reactions were carried out as described in the Methods section.

In contrast to the proteins just discussed, TopBP1 did not show any preference for any particular DNA structure under these reaction conditions, indicating that it cannot discriminate between these different forms of DNA under low-stringency conditions (**Figure 5A**). Cdc45, a target of the ATR-Chk1 intra-S phase DNA damage checkpoint response, did not associate with any DNA structure under a variety of conditions tested (**Figure 5B**).

### Association of ATR-Chk1 pathway proteins with bulky DNA adducts

We next investigated the association of our panel of ATR-Chk1 checkpoint pathway proteins with DNA treated with *N*-acetoxy-2-acetylaminofluorene (AAF), a potent carcinogen that forms bulky adducts on the C8 position of guanine. In these experiments, we used undamaged and AAF-damaged plasmid DNA immobilized on streptavidin-coupled magnetic beads. Importantly, since both ends of the plasmid were biotinylated, there were no free DNA ends for proteins to recognize; thus, the association of proteins with DNA in these experiments indicates a direct association with either undamaged or damaged duplex DNA.

**Figure 6:**
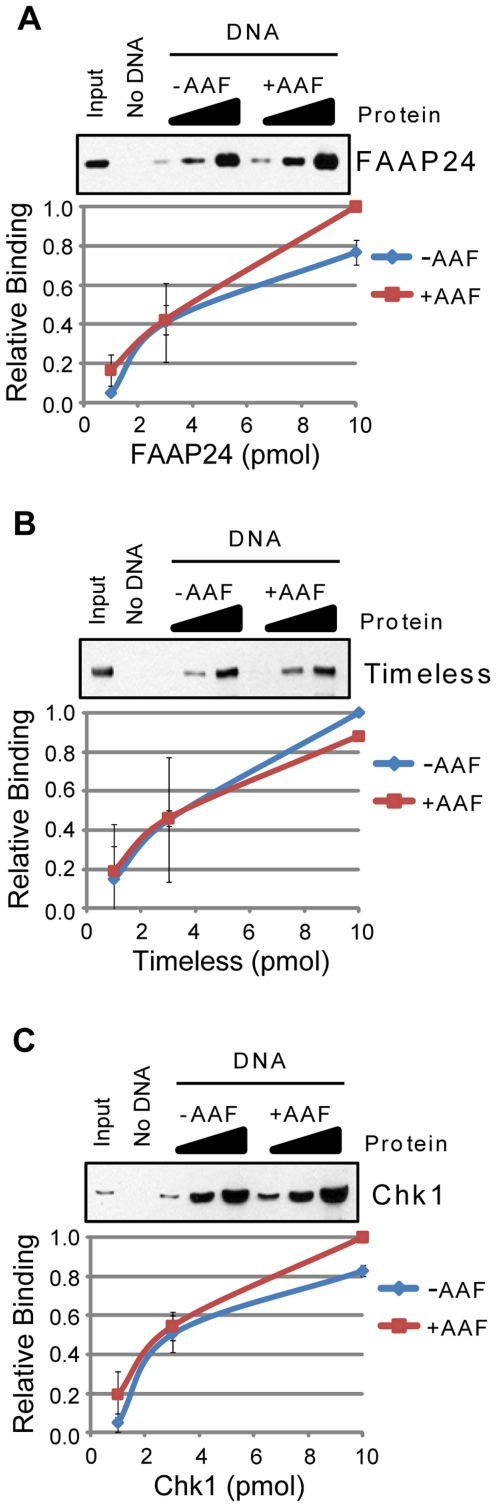
FAAP24, Timeless, and Chk1 do not preferentially bind AAF-damaged DNA. The association of each of the indicated purified proteins with beads alone (No DNA) or to undamaged (−AAF) or *N*-acetoxy-2-acetylaminofluorene (AAF)-treated (+AAF) plasmid DNA bound to magnetic beads was analyzed by SDS-PAGE and immunoblotting. Each of the following proteins was analyzed for binding to each DNA structure: (A) FAAP24, (B) Timeless, and (C) Chk1. Reactions were carried out as described in the Methods section.

**Figure 7:**
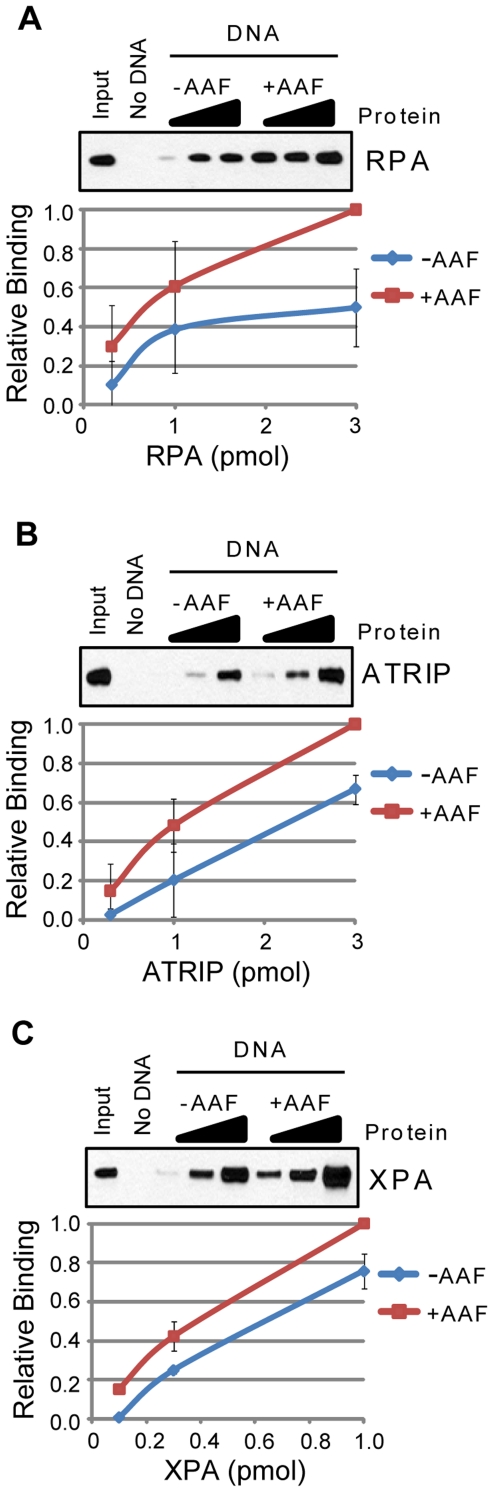
RPA, ATRIP, and XPA show a slight preference for AAF-damaged DNA. The association of each of the indicated purified proteins with beads alone (No DNA) or to undamaged (−AAF) or *N*-acetoxy-2-acetylaminofluorene (AAF)-treated (+AAF) plasmid DNA bound to magnetic beads was analyzed by SDS-PAGE and immunoblotting. Each of the following proteins was analyzed for binding to each DNA structure: (A) RPA, (B) ATRIP, and (C) XPA. Reactions were carried out as described in the Methods section.

As shown in **Figure 6**, FAAP24 (Figure 6A), Timeless (Figure 6B), and Chk1 (Figure 6C) were unable to differentiate between the two DNA substrates under these binding conditions. In contrast, as shown in **Figure 7**, other proteins, including RPA (Figure 7A), ATRIP (Figure 7B), and XPA (Figure 7C) showed a small, less than 2-fold preference for the bulky adduct-containing DNA. Using alternative approaches and techniques, such as EMSA, similar results have previously been observed for RPA and XPA [49]. These results indicate that alone, neither of these two factors can clearly discriminate damaged from undamaged DNA. Our finding that ATRIP showed a small preference for AAF-damaged DNA (Figure 7B) suggests that previous reports of ATR kinase stimulation by bulky adduct-containing DNA [9], [10], [29] may be due in part to an interaction of ATRIP with damaged DNA.

**Figure 8:**
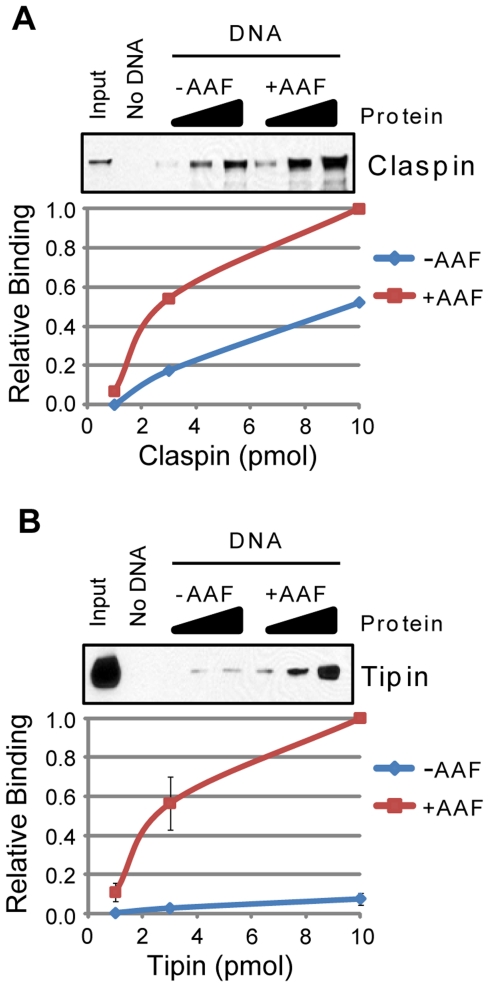
Claspin and Tipin preferentially bind to AAF-damaged DNA. The association of each of the indicated purified proteins with beads alone (No DNA) or to undamaged (−AAF) or *N*-acetoxy-2-acetylaminofluorene (AAF)-treated (+AAF) plasmid DNA bound to magnetic beads was analyzed by SDS-PAGE and immunoblotting. Each of the following proteins was analyzed for binding to each DNA structure: (A) Claspin and (B) Tipin. Reactions were carried out as described in the Methods section.

Interestingly, as shown in **Figure 8**, both Claspin (Figure 8A) and Tipin (Figure 8B) showed a stronger association with the AAF-damaged DNA than the undamaged DNA. Analysis of fragments of these proteins identified smaller domains that are sufficient for this characteristic binding property (Figure S1). Though both of these factors mediate Chk1 phosphorylation by ATR in response to UV and UV-mimetic agents [21], [31], [39], [50], there has previously been no evidence that these proteins directly recognize bulky DNA adducts. However, since both Claspin and Tipin showed increased affinity for the branched DNA structure (Figure 4) in comparison to either ssDNA or dsDNA, these results indicate that the recognition of multiple checkpoint-inducing DNA structures by these proteins may contribute to their DNA damage checkpoint functions.

### Ionic strength affects protein association with bulky adduct-containing DNA

**Figure 9:**
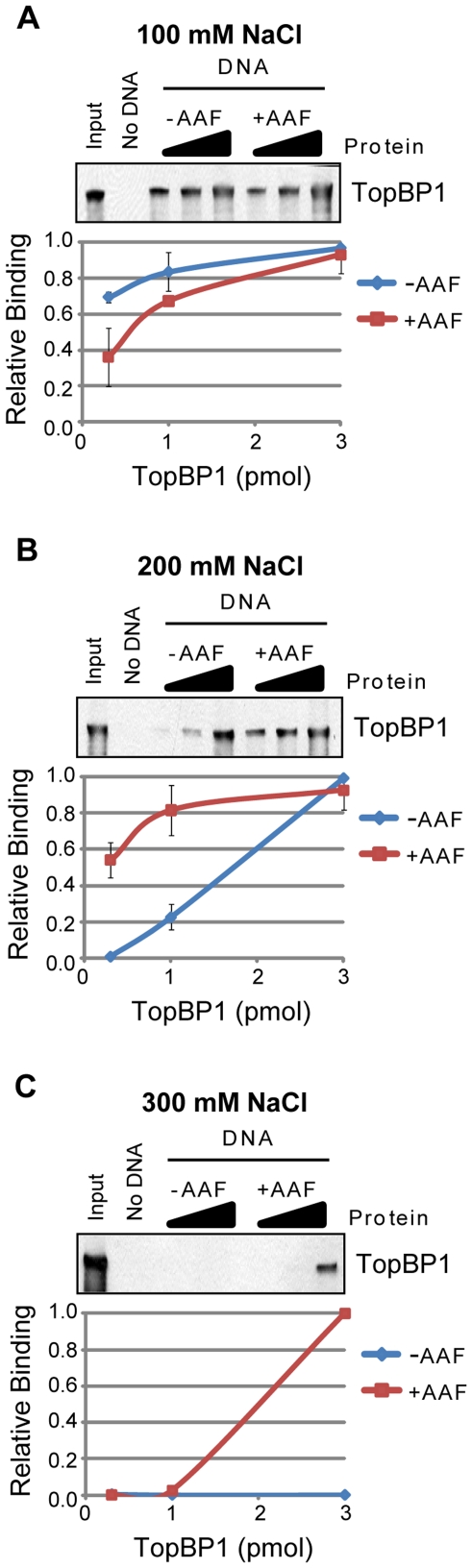
Effect of ionic strength on discrimination of damaged and undamaged DNA. The association of each of TopBP1 with beads alone (No DNA) or to undamaged (−AAF) or AAF-damaged (+AAF) plasmid DNA bound to magnetic beads was analyzed by SDS-PAGE and immunoblotting. Binding reactions contained either (A) 100 mM NaCl, (B) 200 mM NaCl, or (C) 300 mM NaCl. Reactions were carried out as described in the Methods section.

Interestingly, as was seen for FAAP24, Timeless, and Chk1 (Figure 6), we observed that TopBP1 did not show preferential binding to AAF-damaged DNA when binding reactions were performed using low-stringency (100 mM NaCl) conditions (**Figure 9A**). However, using an alternative approach with immobilized protein and radiolabeled damaged DNA, TopBP1 was previously shown to preferentially bind to DNA containing bulky AAF and benzo[a]pyrene diol epoxide (BPDE) adducts [9], [10]. Importantly, the damaged DNA-binding properties of TopBP1 was shown to be functionally relevant in these studies because it correlated with the ability of TopBP1 to mediate damaged DNA-dependent stimulation of ATR in *in vitro* kinase reactions [9], [10].

To reconcile these apparently contradictory results, we tested whether the association of TopBP1 with DNA was sensitive to the ionic strength of the binding reaction. We therefore repeated the pull-down experiments with undamaged and AAF-damaged DNA but instead used higher salt concentrations in the binding reaction and wash steps. As shown in **Figure 9B**, in reactions containing 200 mM NaCl (vs. 100 mM in Figure 9A), TopBP1showed elevated binding to the AAF-damaged DNA at the two lowest protein concentrations. Furthermore, at 300 mM NaCl, TopBP1 was only retained on the AAF-damaged DNA at its highest concentration (**Figure 9C**). Together, these results indicate that TopBP1 is capable of preferentially binding to damaged DNA; however, this binding preference requires more stringent conditions (higher ionic strength). We predict that other proteins that show limited discrimination at lower ionic strengths may similarly show this property.

## Discussion

The diversity and number of protein-DNA interactions that are involved in activating the ATR-Chk1 pathway in response to DNA damage and replication stress remain unclear. Based on a variety of genetic, biochemical, and cell biological approaches, strong evidence supports the notion that ssDNA and primer-template junctions are two primary components of ATR activation [4]. Through recruitment of ATR-ATRIP to ssDNA by RPA and the recruitment of the ATR activator TopBP1 to primer-template junctions by the 9-1-1 clamp, ATR is thought to become fully activated towards checkpoint substrates such as Chk1. Consistent with this suggested model, experiments utilizing *Xenopus* egg extracts [51] and purified yeast and human checkpoint factors [52]–[54] have indeed shown that these proteins and DNA structures constitute a minimal set of factors that can activate ATR.

However, additional data suggest that these factors are not necessarily essential for ATR-Chk1 pathway activation *in vivo* and that alternative mechanisms for stimulation may exist. For example, mutation of ATRIP such that it is unable to stably bind RPA or be recruited to sub-nuclear DNA damage foci does not significantly affect DNA-damaged induced phosphorylation of Chk1 in human cells [7], [35]. Similarly, in *Saccharomyces cerevisiae* cells exposed during S phase to UV-mimetics or the replication inhibitor hydroxyurea, the checkpoint transducing kinase Rad53 becomes phosphorylated in a Mec1 (ATR homolog)-dependent but Dpb11 (TopBP1 homolog)- and Ddc1 (Rad9 homolog)-independent manner [53]. Furthermore, ATR may become activated through the direct recognition of DNA damage by ATR alone [8] or in conjunction with the activating protein TopBP1 [9], [10]. Lastly, a variety of DNA repair proteins directly or indirectly associate with ATR and other checkpoint pathway proteins [16]–[18], [36]; these interactions may therefore facilitate the phosphorylation of checkpoint substrates by ATR.

In this report we systematically analyzed the association of human ATR-Chk1 checkpoint pathway proteins with several checkpoint-inducing DNA structures, including ssDNA, branched DNA, and bulky adduct-containing DNA. We found that many of these proteins showed a greater association with these checkpoint-inducing DNA structures than with undamaged, double-stranded DNA. Furthermore, we also showed that the ionic strength of the reaction greatly influenced the relative association of proteins with damaged and undamaged DNA. Since reconstituted systems composed of only purified ATR-ATRIP, TopBP1, and Chk1 are capable of stimulating Chk1 phosphorylation in response to bulky adduct-containing DNA [9], [10], [29], we believe that a subset of the protein-DNA interactions we observed (i.e., TopBP1 and ATRIP) are functionally relevant.

Interestingly, under conditions of modest ionic strength, we discovered that the checkpoint mediator proteins Tipin and Claspin showed the strongest preferential association with checkpoint-inducing DNA structures (Figures 4, 8). The branched DNA binding data in particular are consistent with chromatin immunoprecipitation experiments in yeast and biochemical fractionation experiments in *Xenopus* egg extracts that show that Tipin and Claspin (and their yeast counterparts) are enriched at normal or stalled replication forks [45], [46], [50], [55]. Though both Claspin and Tipin mediate Chk1 phosphorylation by ATR in response to UV and UV-mimetic agents [21], [39], [50], whether the protein-DNA interactions described here are important for the function of these proteins in ATR-Chk1 signaling *in vivo* is not known. Additional experiments are therefore necessary to clarify the importance of these protein-DNA interactions.

We conclude from these results that there are many protein-DNA interactions that may be important for association and accumulation of ATR-Chk1 pathway proteins at sites of DNA damage and replication stress. However, we recognize and note that only limited conclusions can be drawn from protein-DNA interaction studies in the absence of additional methods and approaches. Whether any or all of the checkpoint protein-DNA interactions we described here are biologically relevant to ATR-Chk1 signaling *in vivo* clearly requires further investigation. The identification of new protein-DNA interactions should aid this process and lead to a greater understanding of the mechanisms of ATR-Chk1 pathway activation in response to DNA damage and replication stress.

## Supporting Information

Figure S1 **Analysis of Timeless, Tipin, and Claspin fragment binding to DNA substrates.** The indicated fragments of (A) Timeless, (B) Tipin, and (C) Claspin were purified from either insect or bacterial expression systems and analyzed for binding to the indicated DNA substrates as described in the Methods section. For each protein, a diagram of the purified protein fragments is provided (top), along with a coomassie-stained gel showing the purified fragments (middle), and immunoblot analyses of protein binding to the DNA substrates (bottom). Input represents 5% of the protein used in the binding reaction.
(TIF)

## References

[1] Reardon JT, Sancar A (2005). Nucleotide excision repair.. Prog Nucleic Acid Res Mol Biol.

[2] Sancar A, Lindsey-Boltz LA, Unsal-Kacmaz K, Linn S (2004). Molecular mechanisms of mammalian DNA repair and the DNA damage checkpoints.. Annu Rev Biochem.

[3] Ciccia A, Elledge SJ (2010). The DNA damage response: Making it safe to play with knives.. Mol Cell.

[4] Cimprich KA, Cortez D (2008). ATR: An essential regulator of genome integrity.. Nat Rev Mol Cell Biol.

[5] Liu P, Barkley LR, Day T, Bi X, Slater DM (2006). The Chk1-mediated S-phase checkpoint targets initiation factor Cdc45 via a Cdc25A/Cdk2-independent mechanism.. J Biol Chem.

[6] Cortez D, Guntuku S, Qin J, Elledge SJ (2001). ATR and ATRIP: Partners in checkpoint signaling.. Science.

[7] Zou L, Elledge SJ (2003). Sensing DNA damage through ATRIP recognition of RPA-ssDNA complexes.. Science.

[8] Unsal-Kacmaz K, Makhov AM, Griffith JD, Sancar A (2002). Preferential binding of ATR protein to UV-damaged DNA.. Proc Natl Acad Sci U S A.

[9] Choi JH, Lindsey-Boltz LA, Sancar A (2009). Cooperative activation of the ATR checkpoint kinase by TopBP1 and damaged DNA.. Nucleic Acids Res.

[10] Choi JH, Lindsey-Boltz LA, Sancar A (2007). Reconstitution of a human ATR-mediated checkpoint response to damaged DNA.. Proc Natl Acad Sci U S A.

[11] Binz SK, Sheehan AM, Wold MS (2004). Replication protein A phosphorylation and the cellular response to DNA damage.. DNA Repair (Amst).

[12] Wold MS (1997). Replication protein A: A heterotrimeric, single-stranded DNA-binding protein required for eukaryotic DNA metabolism.. Annu Rev Biochem.

[13] Delacroix S, Wagner JM, Kobayashi M, Yamamoto K, Karnitz LM (2007). The Rad9-Hus1-Rad1 (9-1-1) clamp activates checkpoint signaling via TopBP1.. Genes Dev.

[14] Collis SJ, Ciccia A, Deans AJ, Horejsi Z, Martin JS (2008). FANCM and FAAP24 function in ATR-mediated checkpoint signaling independently of the fanconi anemia core complex.. Mol Cell.

[15] Huang M, Kim JM, Shiotani B, Yang K, Zou L (2010). The FANCM/FAAP24 complex is required for the DNA interstrand crosslink-induced checkpoint response.. Mol Cell.

[16] Yoshioka K, Yoshioka Y, Hsieh P (2006). ATR kinase activation mediated by MutSalpha and MutLalpha in response to cytotoxic O6-methylguanine adducts.. Mol Cell.

[17] Liu Y, Fang Y, Shao H, Lindsey-Boltz L, Sancar A (2010). Interactions of human mismatch repair proteins MutSalpha and MutLalpha with proteins of the ATR-Chk1 pathway.. J Biol Chem.

[18] Wang Y, Qin J (2003). MSH2 and ATR form a signaling module and regulate two branches of the damage response to DNA methylation.. Proc Natl Acad Sci U S A.

[19] Kumagai A, Lee J, Yoo HY, Dunphy WG (2006). TopBP1 activates the ATR-ATRIP complex.. Cell.

[20] Kumagai A, Kim SM, Dunphy WG (2004). Claspin and the activated form of ATR-ATRIP collaborate in the activation of Chk1.. J Biol Chem.

[21] Chini CC, Chen J (2003). Human claspin is required for replication checkpoint control.. J Biol Chem.

[22] Kemp MG, Akan Z, Yilmaz S, Grillo M, Smith-Roe SL (2010). Tipin-RPA interaction mediates Chk1 phosphorylation by ATR in response to genotoxic stress.. J Biol Chem.

[23] Bonilla CY, Melo JA, Toczyski DP (2008). Colocalization of sensors is sufficient to activate the DNA damage checkpoint in the absence of damage.. Mol Cell.

[24] Lindsey-Boltz LA, Sancar A (2011). Tethering DNA damage checkpoint mediator proteins topoisomerase II{beta}-binding protein 1 (TopBP1) and claspin to DNA activates ataxia-telangiectasia mutated and RAD3-related (ATR) phosphorylation of checkpoint kinase 1 (Chk1).. J Biol Chem.

[25] Soutoglou E, Misteli T (2008). Activation of the cellular DNA damage response in the absence of DNA lesions.. Science.

[26] Henricksen LA, Umbricht CB, Wold MS (1994). Recombinant replication protein A: Expression, complex formation, and functional characterization.. J Biol Chem.

[27] Lindsey-Boltz LA, Bermudez VP, Hurwitz J, Sancar A (2001). Purification and characterization of human DNA damage checkpoint rad complexes.. Proc Natl Acad Sci U S A.

[28] Unsal-Kacmaz K, Sancar A (2004). Quaternary structure of ATR and effects of ATRIP and replication protein A on its DNA binding and kinase activities.. Mol Cell Biol.

[29] Choi JH, Sancar A, Lindsey-Boltz LA (2009). The human ATR-mediated DNA damage checkpoint in a reconstituted system.. Methods.

[30] Sar F, Lindsey-Boltz LA, Subramanian D, Croteau DL, Hutsell SQ (2004). Human claspin is a ring-shaped DNA-binding protein with high affinity to branched DNA structures.. J Biol Chem.

[31] Unsal-Kacmaz K, Chastain PD, Qu PP, Minoo P, Cordeiro-Stone M (2007). The human Tim/Tipin complex coordinates an intra-S checkpoint response to UV that slows replication fork displacement.. Mol Cell Biol.

[32] Sercin O, Kemp MG (2011). Characterization of functional domains in human claspin.. Cell Cycle.

[33] Reardon JT, Sancar A (2006). Purification and characterization of escherichia coli and human nucleotide excision repair enzyme systems.. Methods Enzymol.

[34] Kemp MG, Mason AC, Carreira A, Reardon JT, Haring SJ (2010). An alternative form of replication protein a expressed in normal human tissues supports DNA repair.. J Biol Chem.

[35] Ball HL, Myers JS, Cortez D (2005). ATRIP binding to replication protein A-single-stranded DNA promotes ATR-ATRIP localization but is dispensable for Chk1 phosphorylation.. Mol Biol Cell.

[36] Shell SM, Li Z, Shkriabai N, Kvaratskhelia M, Brosey C (2009). Checkpoint kinase ATR promotes nucleotide excision repair of UV-induced DNA damage via physical interaction with xeroderma pigmentosum group A.. J Biol Chem.

[37] Bomgarden RD, Lupardus PJ, Soni DV, Yee MC, Ford JM (2006). Opposing effects of the UV lesion repair protein XPA and UV bypass polymerase eta on ATR checkpoint signaling.. EMBO J.

[38] Kumagai A, Dunphy WG (2000). Claspin, a novel protein required for the activation of Chk1 during a DNA replication checkpoint response in xenopus egg extracts.. Mol Cell.

[39] Unsal-Kacmaz K, Mullen TE, Kaufmann WK, Sancar A (2005). Coupling of human circadian and cell cycles by the timeless protein.. Mol Cell Biol.

[40] Byun TS, Pacek M, Yee MC, Walter JC, Cimprich KA (2005). Functional uncoupling of MCM helicase and DNA polymerase activities activates the ATR-dependent checkpoint.. Genes Dev.

[41] Parrilla-Castellar ER, Arlander SJ, Karnitz L (2004). Dial 9-1-1 for DNA damage: The Rad9-Hus1-Rad1 (9-1-1) clamp complex.. DNA Repair (Amst).

[42] Bomgarden RD, Yean D, Yee MC, Cimprich KA (2004). A novel protein activity mediates DNA binding of an ATR-ATRIP complex.. J Biol Chem.

[43] Yang Z, Roginskaya M, Colis LC, Basu AK, Shell SM (2006). Specific and efficient binding of xeroderma pigmentosum complementation group A to double-strand/single-strand DNA junctions with 3′- and/or 5′-ssDNA branches.. Biochemistry.

[44] Noguchi E, Noguchi C, McDonald WH, Yates JR, Russell P (2004). Swi1 and Swi3 are components of a replication fork protection complex in fission yeast.. Mol Cell Biol.

[45] Bando M, Katou Y, Komata M, Tanaka H, Itoh T (2009). Csm3, Tof1, and Mrc1 form a heterotrimeric mediator complex that associates with DNA replication forks.. J Biol Chem.

[46] Katou Y, Kanoh Y, Bando M, Noguchi H, Tanaka H (2003). S-phase checkpoint proteins Tof1 and Mrc1 form a stable replication-pausing complex.. Nature.

[47] Tanaka T, Yokoyama M, Matsumoto S, Fukatsu R, You Z (2010). Fission yeast Swi1-Swi3 complex facilitates DNA binding of Mrc1.. J Biol Chem.

[48] Zhao H, Russell P (2004). DNA binding domain in the replication checkpoint protein Mrc1 of schizosaccharomyces pombe.. J Biol Chem.

[49] Reardon JT, Sancar A (2003). Recognition and repair of the cyclobutane thymine dimer, a major cause of skin cancers, by the human excision nuclease.. Genes Dev.

[50] Lee J, Kumagai A, Dunphy WG (2003). Claspin, a Chk1-regulatory protein, monitors DNA replication on chromatin independently of RPA, ATR, and Rad17.. Mol Cell.

[51] MacDougall CA, Byun TS, Van C, Yee MC, Cimprich KA (2007). The structural determinants of checkpoint activation.. Genes Dev.

[52] Majka J, Niedziela-Majka A, Burgers PM (2006). The checkpoint clamp activates Mec1 kinase during initiation of the DNA damage checkpoint.. Mol Cell.

[53] Navadgi-Patil VM, Burgers PM (2009). The unstructured C-terminal tail of the 9-1-1 clamp subunit Ddc1 activates Mec1/ATR via two distinct mechanisms.. Mol Cell.

[54] Choi JH, Lindsey-Boltz LA, Kemp M, Mason AC, Wold MS (2010). Reconstitution of RPA-covered single-stranded DNA-activated ATR-Chk1 signaling.. Proc Natl Acad Sci U S A.

[55] Errico A, Costanzo V, Hunt T (2007). Tipin is required for stalled replication forks to resume DNA replication after removal of aphidicolin in xenopus egg extracts.. Proc Natl Acad Sci U S A.

