# Supplementary figures and images for: Multiple ATR-Chk1 Pathway Proteins Preferentially Associate with Checkpoint-Inducing DNA Substrates
Source: PLoS One. 2011 Jul 29;6(7):e22986. doi: 10.1371/journal.pone.0022986 (PMC3146532; doi:10.1371/journal.pone.0022986)

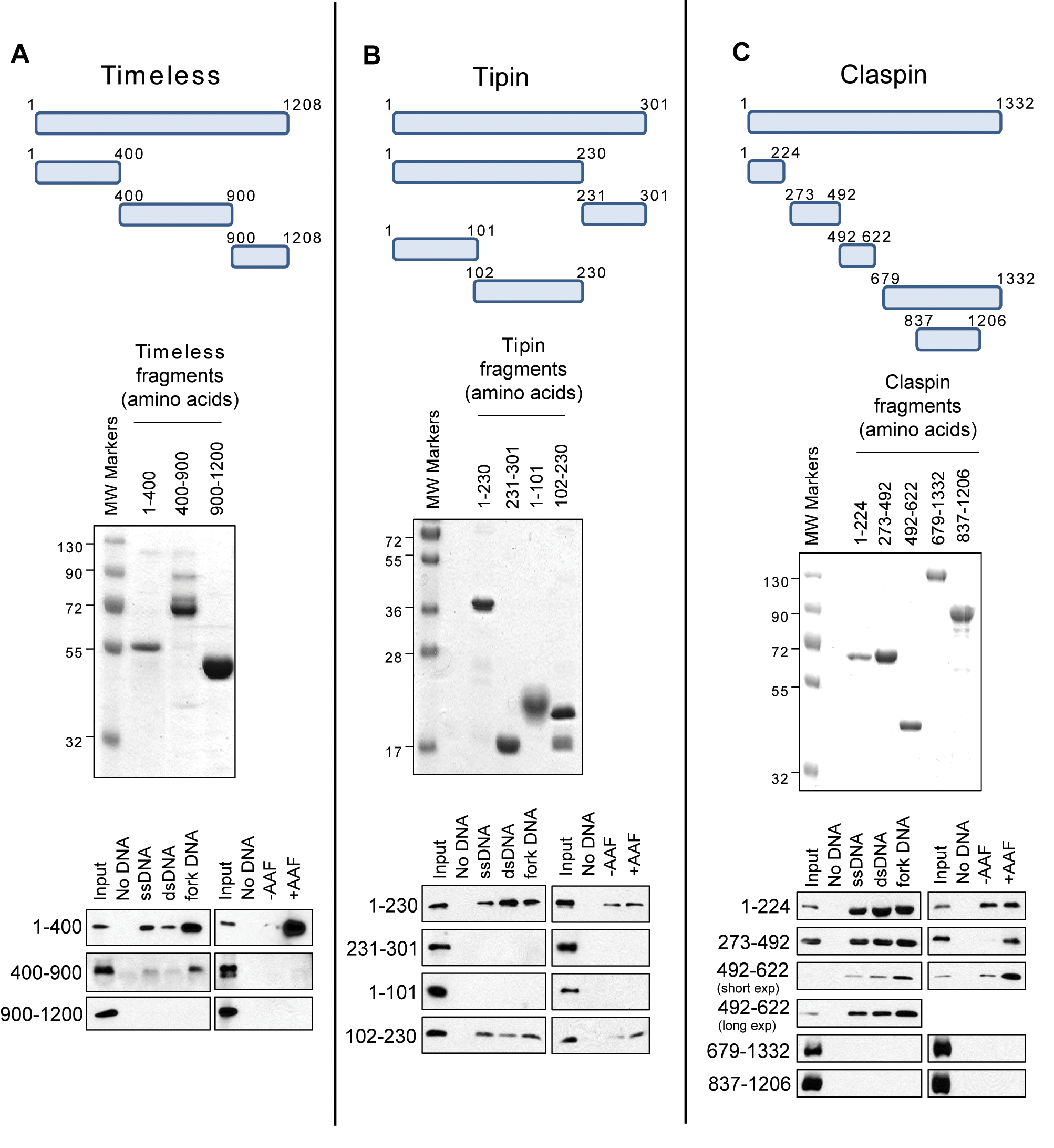

Supplement: Figure S1 — Analysis of Timeless, Tipin, and Claspin fragment binding to DNA substrates. The indicated fragments of (A) Timeless, (B) Tipin, and (C) Claspin were purified from either insect or bacterial expression systems and analyzed for binding to the indicated DNA substrates as described in the Methods section. For each protein, a diagram of the purified protein fragments is provided (top), along with a coomassie-stained gel showing the purified fragments (middle), and immunoblot analyses of protein binding to the DNA substrates (bottom). Input represents 5% of the protein used in the binding reaction. (TIF) [file pone.0022986.s001.tif]
